# Decoding lymphomyeloid divergence and immune hyporesponsiveness in G-CSF-primed human bone marrow by single-cell RNA-seq

**DOI:** 10.1038/s41421-022-00417-y

**Published:** 2022-06-22

**Authors:** Guoju You, Man Zhang, Zhilei Bian, Huidong Guo, Zhengyang Xu, Yanli Ni, Yu Lan, Wen Yue, Yandong Gong, Yingjun Chang, Xiaojun Huang, Bing Liu

**Affiliations:** 1 State Key Laboratory of Primate Biomedical Research, State Key Laboratory of Experimental Hematology, School of Medicine, Tsinghua University, Beijing, China; 2 State Key Laboratory of Proteomics, Academy of Military Medical Sciences, Academy of Military Sciences, Beijing, China; 3 Key Laboratory for Regenerative Medicine of Ministry of Education, Institute of Hematology, School of Medicine, Jinan University, Guangzhou, Guangdong China; 4 Department of Hematology, the First Affiliated Hospital of Zhengzhou University, Academy of Medical Sciences, Zhengzhou University, Zhengzhou, Henan China; 5 Peking University People’s Hospital & Peking University Institute of Hematology, National Clinical Research Center for Hematologic Disease, Beijing Key Laboratory of Hematopoietic Stem Cell Transplantation, Beijing, China; 6 State Key Laboratory of Experimental Hematology, State Key Laboratory of Primate Biomedical Research, Institute of Hematology, Fifth Medical Center of Chinese PLA General Hospital, Beijing, China; 7 Stem Cell and Regenerative Medicine Lab, Institute of Health Service and Transfusion Medicine, Academy of Military Medical Sciences, Beijing, China; 8 South China Research Center for Stem Cell & Regenerative Medicine, Guangzhou, Guangdong China; 9 State Key Laboratory of Experimental Hematology, Haihe Laboratory of Cell Ecosystem, Institute of Hematology, Fifth Medical Center of Chinese PLA General Hospital, Beijing, China

**Keywords:** Haematopoietic stem cells, Immunology

## Abstract

Granulocyte colony-stimulating factor (G-CSF) has been widely used to mobilize bone marrow hematopoietic stem/progenitor cells for transplantation in the treatment of hematological malignancies for decades. Additionally, G-CSF is also accepted as an essential mediator in immune regulation, leading to reduced graft-versus-host disease following transplantation. Despite the important clinical roles of G-CSF, a comprehensive, unbiased, and high-resolution survey into the cellular and molecular ecosystem of the human G-CSF-primed bone marrow (G-BM) is lacking so far. Here, we employed single-cell RNA sequencing to profile hematopoietic cells in human bone marrow from two healthy donors before and after 5-day G-CSF administration. Through unbiased bioinformatics analysis, our data systematically showed the alterations in the transcriptional landscape of hematopoietic cells in G-BM, and revealed that G-CSF-induced myeloid-biased differentiation initiated from the stage of lymphoid-primed multipotent progenitors. We also illustrated the cellular and molecular basis of hyporesponsiveness of T cells and natural killer (NK) cells caused by G-CSF stimulation, including the potential direct mechanisms and indirect regulations mediated by ligand–receptor interactions. Taken together, our data extend the understanding of lymphomyeloid divergence and potential mechanisms involved in hyporesponsiveness of T and NK cells in human G-BM, which might provide basis for optimization of stem cell transplantation in hematological malignancy treatment.

## Introduction

Allogeneic hematopoietic stem cell transplantation (allo-HSCT) is a potentially curative approach available for hematological malignancies^1^, but its broad application has been limited by the availability of human leukocyte antigen (HLA)-matched donors and severe graft-versus-host disease (GVHD). In the past ten years, unmanipulated haploidentical blood and marrow transplantation that combines granulocyte colony-stimulating factor (G-CSF)-primed bone marrow (G-BM) and G-CSF-mobilized peripheral blood stem cells (G-PBSC) without in vitro T-cell depletion, has been proved to be a reliable protocol with a low risk of GVHD^2–6^.

As a commonly used mobilizer, G-CSF is known to increase the numbers of hematopoietic stem/progenitor cells (HSPCs) harvested within the bone marrow (BM) and peripheral blood (PB), and lead to enhanced myeloid hematopoiesis and granulopoiesis^7,8^. However, it remains controversial about the mechanisms by which G-CSF regulates hematopoiesis^9^. Bernitz et al.^10^ found that G-CSF treatment could increase the number of myeloid-biased CD41^+^ hematopoietic stem cells (HSCs) in mouse BM. Recently, Xie et al.^11^ showed that G-CSF directly acted on lymphoid-biased HSCs to increase their divisions and maintain their repopulating activity after transplantation using functional experiments at the single-cell level in mice. In addition, due to technical limitations, our understanding of the effect of G-CSF on hematopoietic differentiation in human BM in vivo is scant.

Moreover, G-CSF mobilization has been demonstrated to reduce GVHD with preservation of the graft-versus-leukemia (GVL) effect^12–14^. A growing body of studies demonstrated that G-CSF could attenuate the reactivity of T and natural killer (NK) cells through inducing T helper 2 (Th2) cell polarization^15^, promoting the generation of regulatory T (Treg) cells^16^, tolerogenic dendritic cells (DCs)^17,18^ and possibly myeloid-derived suppressor cells (MDSCs)^19–21^. Intriguingly, compared with G-PBSC transplantation, G-BM transplantation resulted in attenuated acute GVHD and comparable engraftment^22^, indicating that G-BM was different from G-CSF-mobilized peripheral blood (G-PB) in terms of immunomodulatory properties. However, the systematical landscape of immune cells and underlying mechanisms of immunoregulation in G-BM have not yet been well established^9,17,23^.

Herein, using single-cell RNA sequencing (scRNA-seq) and unbiased bioinformatics analysis, we comprehensively characterized the cellular and molecular alterations in the BM from healthy donors upon G-CSF administration and revealed the potential mechanisms of hematopoiesis changes and immune hyporesponsiveness in T and NK cells.

## Results

### Single-cell transcriptomics of hematopoietic cells in human BM upon G-CSF treatment

**Fig. 1:**
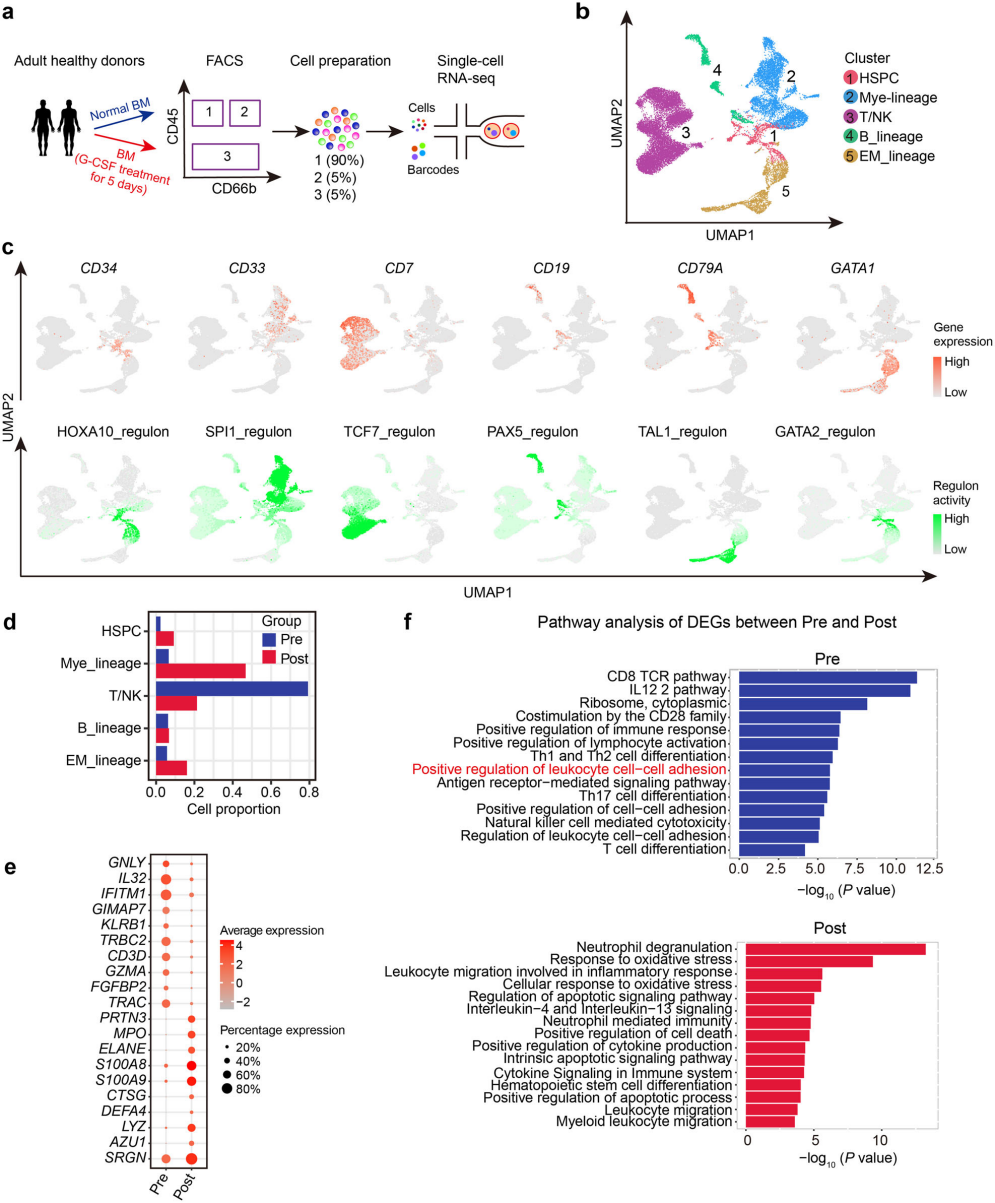
Transcriptomic landscape of hematopoietic cells in human G-BM. **a** Workflow showing the processing of paired unstimulated BM and G-CSF-primed BM (G-BM) samples from healthy donors for scRNA-seq. **b** Uniform manifold approximation and projection (UMAP) visualization of main lineages in human BM based on single-cell transcriptomes. Each dot represents a single cell; colors indicate cell clusters with numbered labels. **c** UMAP visualization of the expression of lineage-specific marker genes (top) and activity of canonical cell type-specific regulons (bottom) in identified cell lineages. **d** Bar plot showing the changes in the percentage of each cell lineage upon G-CSF treatment. Pre, representing samples before G-CSF treatment; Post, representing samples after G-CSF treatment. **e** Dot plot showing average expression of top 10 DEGs for hematopoietic cells in unstimulated BM and G-BM and the percentage of cells expressing the genes. DEGs were detected using function FindAllMarkers in Seurat (Wilcoxon-rank-sum test, with *P* value adjusted for multiple testing using Benjamini–Hochberg correction). **f** Bar plots from the Metascape analysis showing the major enriched terms of DEGs before (top) and after (bottom) G-CSF administration. The length of each bar represents –log_10_(*P* value).

To generate a single-cell transcriptome map of cells in G-BM, we collected BM samples from two healthy donors before and after 5-day G-CSF treatment, and used fluorescence-activated cell sorting (FACS) to isolate different cell lineages and mixed them in a certain ratio for subsequent scRNA-seq (Fig. 1a; Supplementary Fig. S1a). After stringent quality control, a total of 21,005 cells were obtained from four BM samples of two donors and further classified into five cell lineages (Fig. 1b; Supplementary Fig. S1b–d). Based on the expression of canonical marker genes and lineage-associated transcriptional factors (Fig. 1c), we defined them as HSPC (*CD34*; HOXA10 regulon), myeloid lineage (*CD33*; SPI1 regulon), T/NK lineage (*CD7;* TCF7 regulon), B_lineage (*CD19*, *CD79A*; PAX5 regulon) and erythroid/megakaryocyte lineage (EM_lineage) (*GATA1*; TAL1 and GATA2 regulon). As expected, G-CSF treatment caused an increase in percentages of HSPCs and myeloid lineage, while a decrease in percentages of T/NK lineage (Fig. 1d), indicating myeloid-biased hematopoiesis in G-BM. This finding was also supported by increased expression of myeloid hematopoiesis-related genes, such as *LYZ*, *S100A8,* and *S100A9*, and decreased expression of T cell differentiation-related genes *CD3D*, *TRAC,* and *IFITM1* (Fig. 1e, f; Supplementary Table S1). Besides, samples from two donors were highly reproducible for cell composition and overall changes in percentage of each cell lineage (Supplementary Fig. S1d, e).

Notably, hematopoietic cells in G-BM exhibited higher expression of protease genes such as *CTSG* and *ELANE* (Supplementary Fig. S1f)^24^. Gene Ontology (GO) term enrichment analysis also presented reduced cell adhesion (Fig. 1f), suggesting that HSPC mobilization in G-BM could be mediated by disruption of cell adhesion and enhanced proteolytic activity as reported in previous studies^25,26^.

### The myeloid-biased hematopoietic differentiation in G-BM

**Fig. 2:**
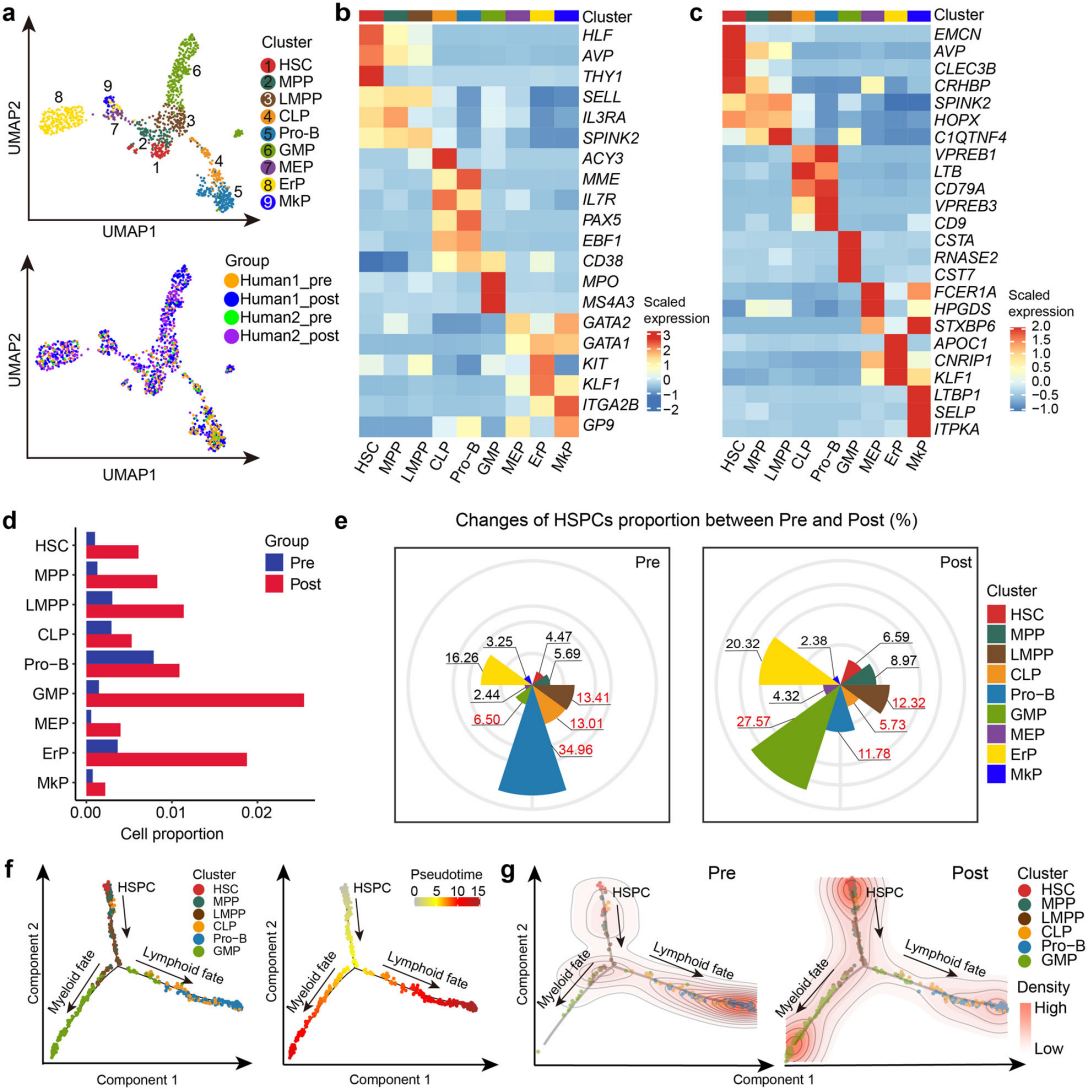
The effect of G-CSF on hematopoietic differentiation in human BM. **a** UMAP visualization of subclusters in HSPCs. Each dot represents a single cell; colors indicate clusters (top) and sources of samples (bottom). HSC hematopoietic stem cell; MPP multipotential progenitor; LMPP lymphoid-primed multipotent progenitor; CLP common lymphocyte progenitor; Pro-B progenitor B cell; GMP granulocyte-monocyte progenitor; MEP megakaryocyte/erythroid progenitor; ErP erythroid progenitor; MkP megakaryocyte progenitor. **b** Heatmap displaying scaled expression of canonical cell type-associated genes for clusters in **a**. Color scale corresponds to z-scored, log-transformed mean gene-expression counts for each cell state. **c** Heatmap showing scaled expression of top 3 DEGs for clusters in **a**. Color scale corresponds to z-scored, log-transformed mean gene-expression counts for each cell state. Detailed DEGs can be found in Supplementary Table S2. **d** Bar plot showing the changes in the percentage of subclusters of HSPCs upon G-CSF treatment. **e** Cell proportion of each subcluster within HSPCs derived from BM grafts before (left) and after (right) G-CSF administration. **f** Monocle prediction of HSPC developmental trajectory with cluster information (left) and pseudotime (right) mapped. **g** Density plots displaying the distribution of different hematopoietic progenitor cells in trajectories before (left) and after (right) G-CSF administration. These plots were based on the 2D kernel density estimation. The density of lines and intensity of colors are proportional to cell density in each state.

We re-clustered the identified HSPCs into nine subpopulations based on the expression of cell type-associated genes, namely HSC, multipotent hematopoietic progenitor (MPP), lymphoid-primed multipotent progenitor (LMPP), common lymphoid progenitor (CLP), progenitor B cell (Pro-B), granulocyte-monocyte progenitor (GMP), megakaryocyte-erythrocyte progenitor (MEP), erythrocyte progenitor (ErP), megakaryocyte progenitor (MkP) (Fig. 2a–c; Supplementary Table S2), all of which underwent an increase in cell percentages upon G-CSF stimulation (Fig. 2d). Interestingly, the mobilized HSC showed a lower proportion in the G1 phase and a higher proportion in the G2M phase, as well as a significantly increased proliferation score (Supplementary Fig. S1g, h), indicating the proliferative status of HSCs in human G-BM, which is consistent with results in mice^10,27^. We further calculated the relative cell proportion within HSPCs. An obviously increased percentage was observed in GMP, which contrasted with the reduced percentage of lymphoid progenitors CLP and Pro-B, suggesting a myeloid-biased differentiation in G-BM (Fig. 2e). However, as the progenitor of CLP and GMP^28^, LMPP showed no obvious change in percentage (Fig. 2e), indicating that LMPP was the earliest target progenitor of G-CSF inducing myeloid-biased hematopoiesis in G-BM. Trajectory inference also showed similar myeloid-biased hematopoiesis bifurcated from LMPP in G-BM (Fig. 2f, g).

### Sub-clustering of T/NK lineage in BM before and after G-CSF administration

**Fig. 3:**
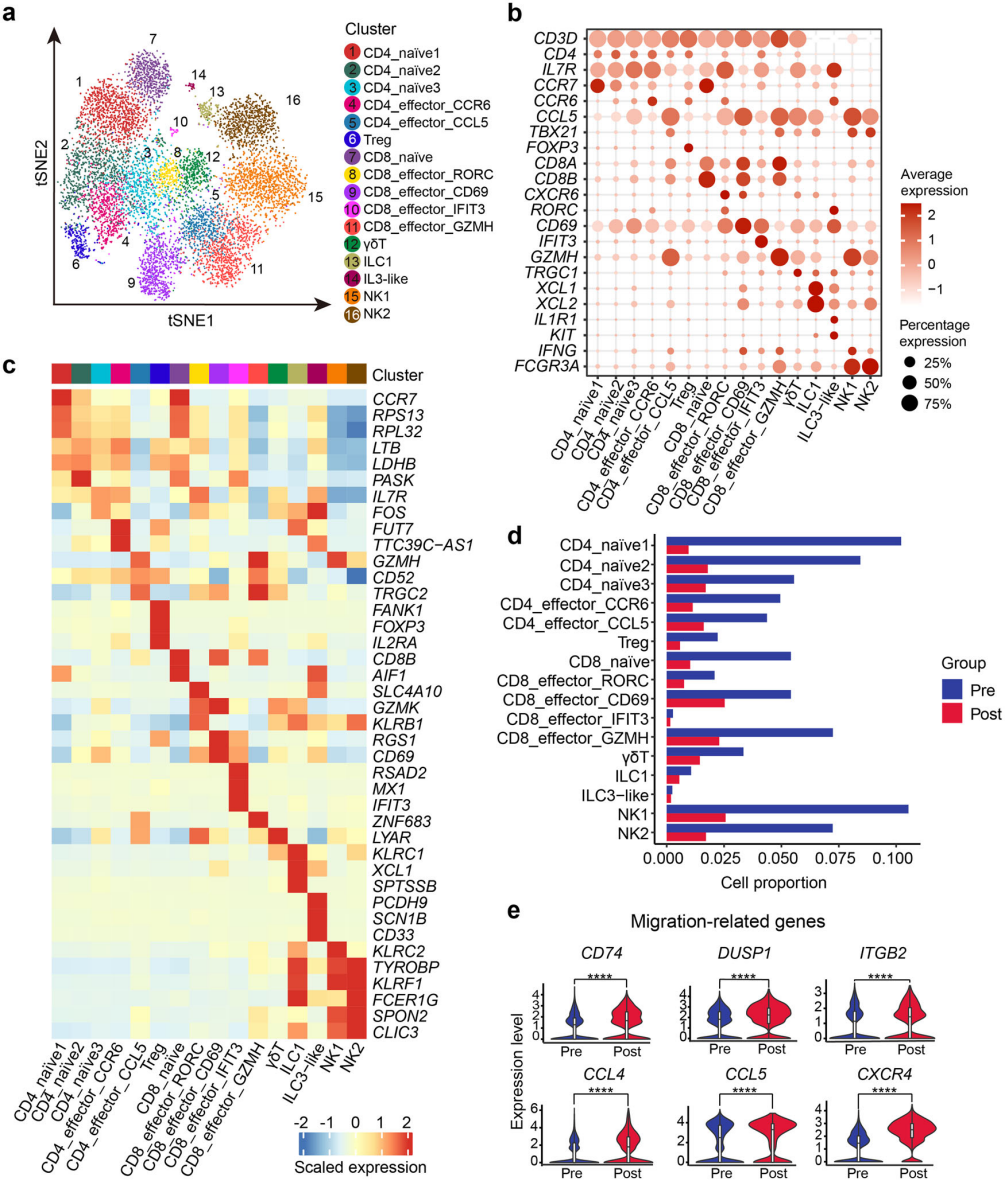
Sub-clustering of T/NK lineage in human BM before and after G-CSF mobilization. **a** t-distributed Stochastic Neighbor Embedding (t-SNE) visualization of 16 subclusters of the T/NK lineage pooled from human BM across two donors. Treg, regulatory T cell; γδT, gamma-delta T cells. **b** Identification of subclusters of T/NK lineage based on the expression of key cell type-associated marker genes. **c** Heatmap showing scaled expression of top 3 DEGs of the subclusters in T/NK cells. Detailed DEGs can be found in Supplementary Table S4. **d** Bar plot displaying the changes in the percentage of various subclusters of T/NK cells upon G-CSF administration. **e** Violin plots showing the changes in expression of genes related to leukocyte migration upon G-CSF administration. Representative upregulated DEGs enriched in leukocyte migration (GO: 0050900) are displayed. ****P value < 0.0001.

To further assess the effect of G-CSF on immune cells, we analyzed the T and NK cell populations. Through unsupervised clustering analysis, a total of 16 subpopulations were identified, including six subtypes of CD4^+^ T cells, five subtypes of CD8^+^ T cells, two subtypes of NK cells, gamma delta T (γδ T) cells, type 1 innate lymphoid cell (ILC1), and type 3 innate lymphoid cell (ILC3)-like cells (Fig. 3a–c; Supplementary Table S3). Among CD4^+^ T cells, we defined three subtypes of naïve CD4^+^ T cells (CD4_naïve1, CD4_naïve2 and CD4_naïve3) and Treg, as well as two effector CD4^+^ T cell subtypes, namely CD4_effector_CCL5 and CD4_effector_CCR6. CD4_effector_CCL5 showed higher expression of T helper 1 (Th1)-related genes (*TBX21* and *IFNG*), while CD4_effector_CCR6 was characterized by higher expression of genes commonly expressed in T helper 17 (Th17) cells (*CCR6*, *FUT7,* and *LTB*). CD8^*+*^ T cells included naïve CD8^+^ T cells (*CD8A*^*+*^*CCR7*^*+*^) and 4 effector CD8^+^ T cell subsets (*CD8A*^+^*CCR7*^–^) exhibiting distinct signatures, namely CD8_effector_CD69, CD8_effector_GZMH, CD8_effector_IFIT3 and CD8_effector_RORC. In detail, CD8_effector_CD69 and CD8_effector_GZMH had higher expression of cytotoxicity-associated genes *GZMK* and *GZMH*, respectively. Intriguingly, *GZMK* (highly expressed by CD8_effector_CD69 and CD8_effector_RORC) and *ZNF683* (highly expressed by CD8_effector_GZMH) were reported to be highly expressed by CD8^+^ T cells in a possible “pre-exhausted” state^29,30^. CD8_effector_IFIT3 was characterized by the expression of genes involved in the type I interferon response (*IFIT3*, *MX1,* and *RSAD2*)^31^ and related receptors (*IFNAR1* and *IFNAR2*) (Fig. 3b, c; Supplementary Fig. S2a), suggesting a role involved in type I interferon response. CD8_effector_RORC with higher expression of *RORC* and *CXCR6*, might be related to IL17-producing CD8^+^ T (Tc17) cells (Fig. 3b, c). Tc17 cells were reported as proinflammatory, plastic pathogenic CD8^+^ T cells that induced GVHD without antileukemic effects^32^. As expected, the percentages of T and NK subpopulations decreased in G-BM (Fig. 3d), which might result from the dilution caused by increased myeloid cells (Fig. 1d). Besides, T and NK cells exhibited higher expression of genes related to migration (Fig. 3e; Supplementary Fig. S2b), which might also contribute to the decrease in the percentage of T/NK lineage.

### Transcriptomic changes of the T/NK lineage in G-BM and the potential direct mechanisms of immunomodulatory effect

**Fig. 4:**
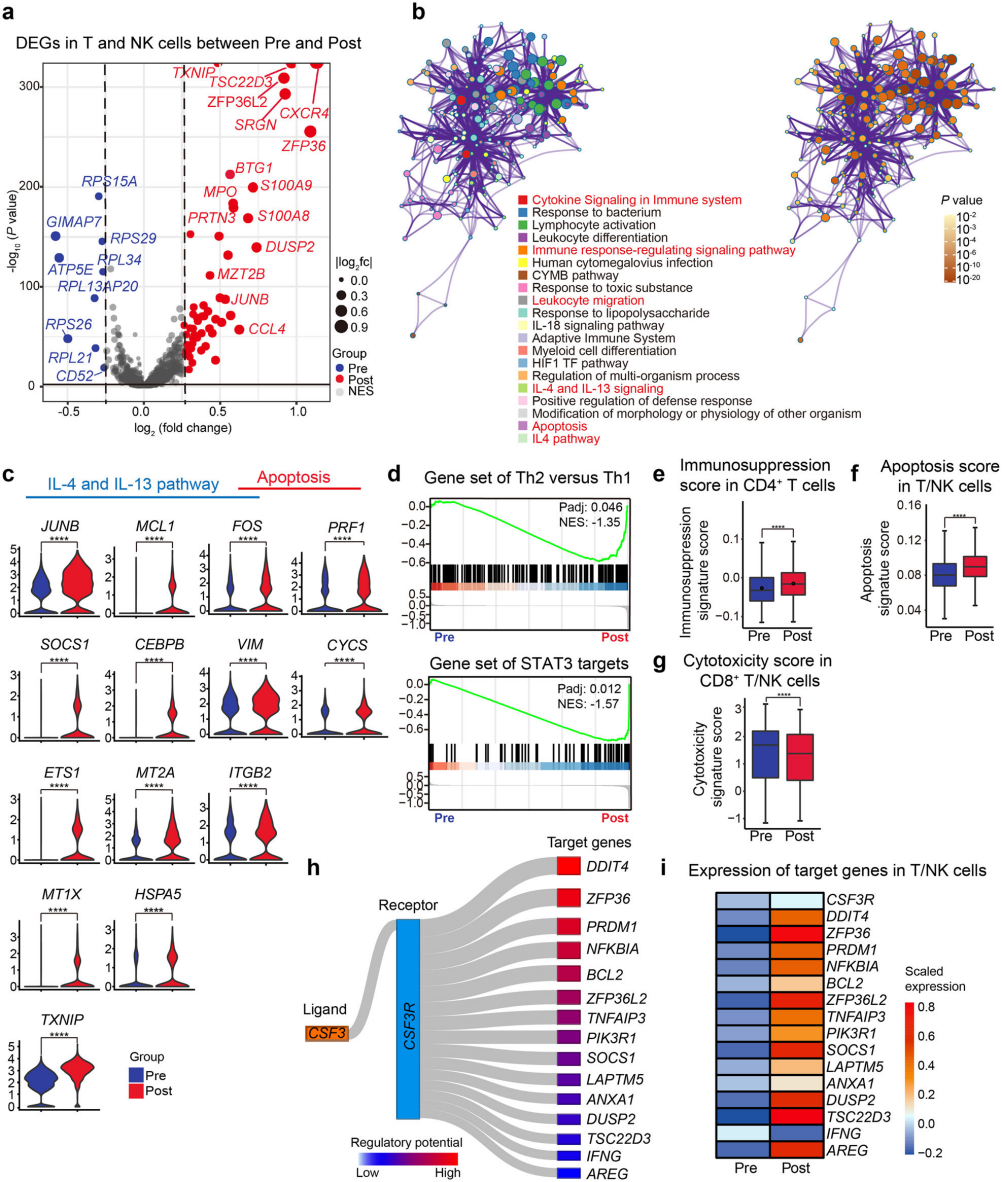
Hyporesponsiveness of T and NK cells induced by G-CSF treatment. **a** Volcano plot showing DEGs of T/NK lineage between unstimulated BM and G-BM. DEGs were detected by differential expression analysis (two-sided Student’s *t*-test). Each dot represents a single gene; genes with log_2_(fold change) (logFC) < −0.25 and logFC > 0.25 were selected and colored in blue and red, respectively. **b** Metascape network enrichment analysis of DEGs in T/NK cells in G-BM, with top 20 enriched terms and corresponding *P* values displayed at the bottom. Nodes were colored to reflect the memberships of enriched terms (left) and statistical *P* values (right). **c** Violin plots displaying the expression of key genes related to the IL-4 and IL-13 pathway, as well as apoptosis in T/NK cells after G-CSF treatment. Representative DEGs enriched in the IL-4 and IL-13 pathway and apoptosis in **b** are displayed. **d** GSEA plots of the enrichment of the Th2 versus Th1 signatures (top) and STAT3 targets (bottom) in CD4^+^ T cells in G-BM compared with unstimulated BM. *P* values adjusted with Benjamini–Hochberg correction and normalized enrichment score (NES) are displayed. **e** Box plot showing immunosuppression scores before and after G-CSF administration in CD4^+^ T cells. *P* values were calculated using the Wilcoxon-rank-sum test. ****P value < 0.0001. **f** Box plot showing the scores of the positive regulation of apoptosis before and after G-CSF administration in T/NK cells. ****P value < 0.0001. **g** Box plot showing the cytotoxicity scores before and after G-CSF administration in CD8^+^ T cells and NK cells. ****P value < 0.0001. **h** Sankey plot showing representative predicted target genes of G-CSF via binding with G-CSFR. Target genes with significantly changed expression are shown. Both the width of connecting lines and the color intensity of boxes represent the regulatory potential. **i** Heatmap showing the expression of *CSF3R* and target genes as presented in **h** in T/NK lineage.

We next elucidated the effects of G-CSF on transcriptomic profiling of T and NK cells in G-BM. In total, the overall differentially expressed gene (DEG) analysis showed upregulated expression of genes related to immune modulation (*DUSP2*^33^ and *TSC22D3*^34^) (Fig. 4a, b; Supplementary Table S4), Th2 developmental pathways (Interleukin (IL)-4 and IL-13 signaling pathway)^35^, and apoptosis (Fig. 4b, c). Gene Set Enrichment Analysis (GSEA) also showed the enrichment of Th2-related signatures in CD4^+^ T cells (Fig. 4d; Supplementary Table S5), which was in line with the previous evidence^36^. Besides, CD4^+^ T cells also displayed a higher enrichment of signal transducer and activator of transcription 3 (STAT3) target genes in G-BM (Fig. 4d), which was required for the Th2 development^37^. In addition, gene scoring analysis showed a significantly elevated immunosuppression score in CD4^+^ T cells (Fig. 4e), and a higher apoptosis score in T/NK cells (Fig. 4f), which further suggests the immunosuppressive features induced by G-CSF.

Upon G-CSF administration, T and NK cells in G-BM unanimously exhibited higher expression of genes related to immunosuppression such as *SOCS1*^38^, *TSC22D3*, *ZFP36*^39^, and *DUSP2* (Supplementary Figs. S2c–h and S3, Table S6). Besides, CD8^+^ T and NK cells exhibited a lower cytotoxicity activity in G-BM (Fig. 4g).

Furthermore, based on ligand–target relationship analysis in NicheNet^40^, the expression of the above upregulated genes related to anti-inflammation (*ZFP36*^39^ and *NFKBIA*^41^) and immunosuppression was predicted to be regulated directly by G-CSF binding with G-CSF receptor (G-CSFR, encoded by *CSF3R*) in T/NK cells^40^, along with slightly higher expression of *CSF3R* following G-CSF stimulation (Fig. 4h, i). G-CSF treatment also reduced the expression of *IFNG* (encoding IFN-γ) in G-BM as previously reported^42,43^, which could result from potential direct regulation by binding of G-CSF with G-CSFR (Fig. 4h, i).

### The effect on myeloid cells of G-CSF in human BM

**Fig. 5:**
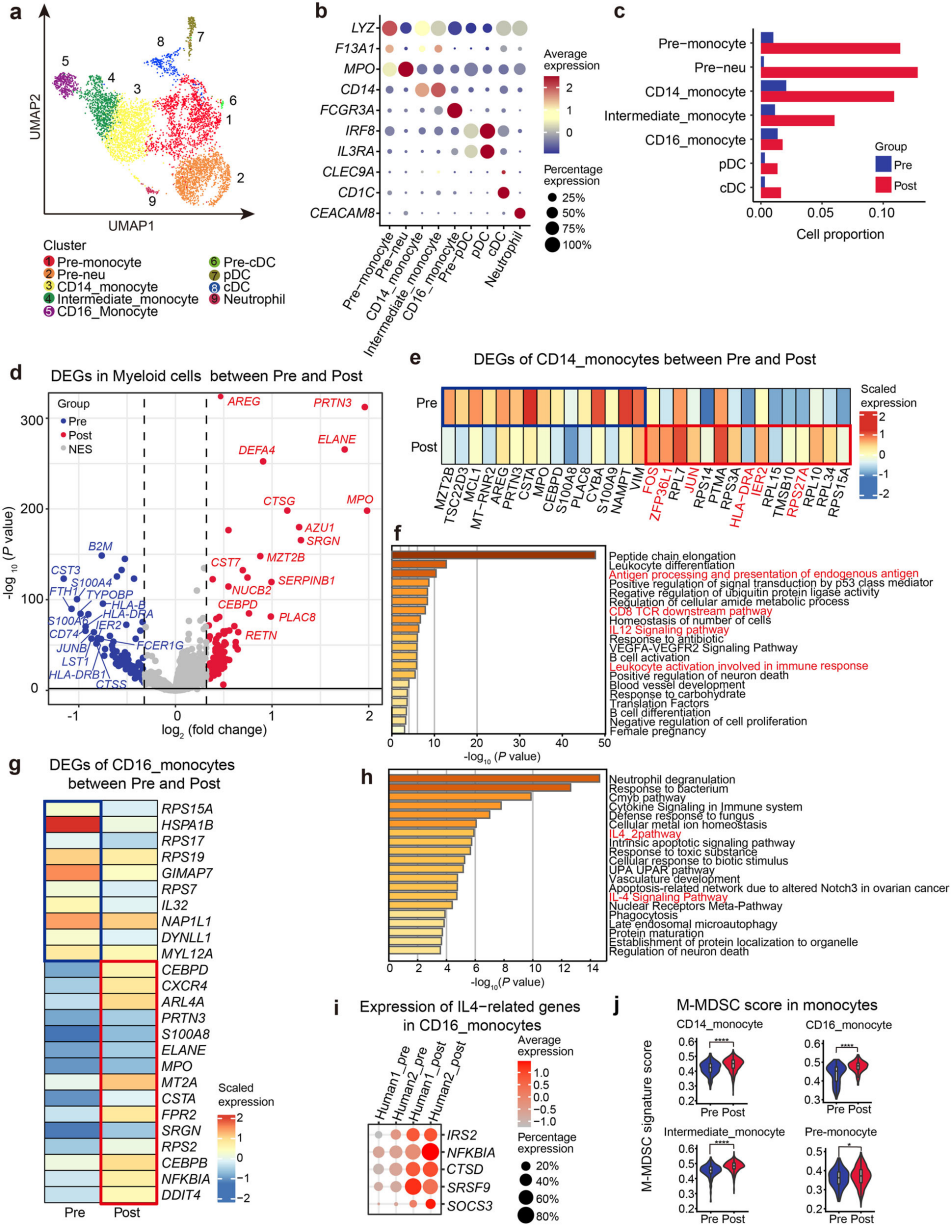
Effect of G-CSF on myeloid cells. **a** UMAP visualization of nine subclusters of the myeloid lineage across two donors. **b** Dot plot showing the average expression of cell type-associated marker genes in myeloid subclusters and percentage of cells expressing these genes. **c** Bar plot displaying changes in the percentage of myeloid subclusters before and after G-CSF treatment. **d** Volcano plot showing the DEGs of myeloid cells in G-BM compared with unstimulated BM. DEGs were detected by differential expression analysis (two-sided Student’s *t*-test). Each dot represents a single gene. Genes with logFC < −0.25 and logFC > 0.25 were selected and colored in blue and red, respectively. **e** Heatmap showing scaled expression of top 15 DEGs in CD14^+^ monocytes before and after G-CSF treatment. **f** Metascape bar graph showing functional enrichment of downregulated genes in CD14^+^ monocytes after G-CSF treatment. Enrichment terms sorted by −log_10_(*P* value) were displayed by bars with a discrete color scale representing statistical significance. The top 20 enriched terms are displayed. **g** Heatmap showing scaled expression of top DEGs in CD16^+^ monocytes after G-CSF treatment. **h** Metascape bar graph showing functional enrichment of upregulated genes in CD16^+^ monocytes after G-CSF treatment. Enrichment terms sorted by −log_10_(*P* value) were displayed by bars with a discrete color scale representing statistical significance. The top 20 enriched terms are shown. **i** Dot plot showing average expression of genes related to IL-4 signaling pathway in CD16^+^ monocytes before and after G-CSF treatment and the percentage of cells expressing these genes. Representative upregulated DEGs enriched in the IL-4 signaling pathway in **h** are displayed. **j** Violin plot showing scores of the M-MDSC signatures before and after G-CSF administration in monocyte subclusters. AddModuleScore function in Seurat R package was used to calculate the average expression with default settings. *P value < 0.05; ****P value < 0.0001.

We further identified subclusters in the myeloid lineage. Myeloid cells contained subclusters such as CD14^+^ monocytes, CD16^+^ monocytes, intermediate monocytes (*CD14*^*+*^*FCGR3A*^*+*^), plasmacytoid dendritic cells (pDCs), conventional dendritic cells (cDCs), neutrophils and two relatively naïve subclusters, named pre-monocyte and pre-neutrophil (Fig. 5a, b; Supplementary Fig. S4a, b and Table S7). With no surprise, the percentage of myeloid lineage cells increased significantly (Fig. 5c), with higher expression of neutrophil degranulation genes (*AZU1* and *MPO*) and lower expression of lymphocyte activation genes (*LST1* and *HLA-DRA*) (Fig. 5d). As the most potent antigen-presenting cells with the ability to coordinate tolerance and immunity among myeloid cells^44^, DCs exhibited higher expression of genes associated with anti-inflammation (*GRN*^45^ and *ZFP36*) and negative regulation of immune response (*TSC22D3* and *SOCS1*) in G-BM (Supplementary Fig. S4c, d and Table S8). It has been described that *SOCS1* highly expressed in DCs could induce T-cell hyporesponsiveness^46,47^. In addition, downregulated pathways in CD14^+^ monocytes included antigen processing and presentation, CD8 TCR downstream pathway (*HLA-DRA* and *IER2*), and proinflammatory IL-12 pathway (*ZFP36L1* and *JUN*) (Fig. 5e, f). CD16^+^ monocytes showed higher expression of genes related to IL-4 signaling pathway (*NFKBIA* and *DDIT4*) (Fig. 5g–i), which is critical in anti-inflammation and Th2 cell differentiation^48,49^. Monocytic-MDSCs (M-MDSCs) have been identified as immature myeloid cells with a remarkable ability to suppress T-cell responses both in antigen-specific and -nonspecific manners associated with the production of nitric oxide and cytokines^50,51^. In our data, all monocyte subclusters exhibited stronger M-MDSC features as indicated by gene scoring analysis using reported MDSC gene signatures (Fig. 5j; Supplementary Table S5)^52^, indicating that G-CSF might modulate M-MDSC generation. Collectively, myeloid cells underwent significant changes in transcriptional features upon G-CSF stimulation, which might contribute to the regulation of T-cell response in multiple ways.

### The potential indirect mechanisms of G-CSF inducing lower reactivity of T and NK cells

**Fig. 6:**
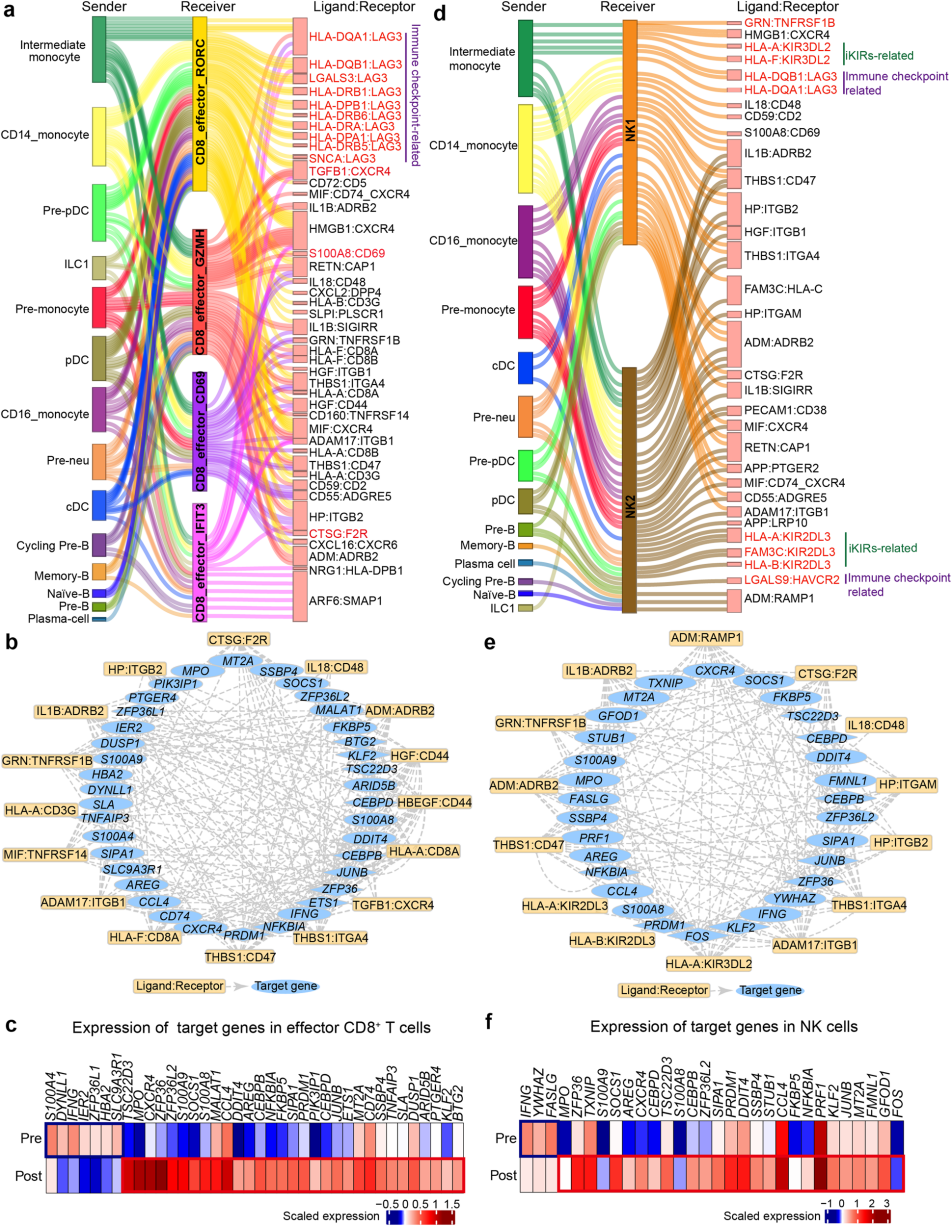
Indirect modulation of G-CSF on T and NK cells. **a** Sankey plot showing the upregulated intercellular communication between the potential sender cells and effector CD8^+^ T cells upon G-CSF administration. Differential intercellular communication analysis was performed using R package scDiffCom. Ligand–receptor pairs with adjusted *P* value < 0.05 and fold change > 1.5 are displayed. **b** Plot showing regulation of top predicted target genes in effector CD8^+^ T cells mediated by upregulated ligand–receptor pairs between effector CD8^+^ T cells and potential sender cells. Transcription factors in target genes are represented as diamonds. The ligand–receptor–target relationship was obtained from the database containing ligand–receptor interactions and receptor–transcription factor interactions in R package scMLnet, and database including ligand–receptor interactions and ligand–target links in R package NicheNet. **c** Heatmap showing the average expression of the downstream target genes in **b** in effector CD8^+^ T cells before and after G-CSF administration. **d** Sankey plot showing the upregulated intercellular communication between the potential sender cells and NK cells upon G-CSF administration. **e** Plot showing the regulation of top predicted target genes in NK cells mediated by upregulated ligand–receptor pairs between NK cells and potential sender cells. **f** Heatmap showing the average expression of the target genes in **e** in NK cells before and after G-CSF administration.

It is generally assumed that T and NK cells can be affected by G-CSF indirectly^42,53^. Thus, we further explored altered cell–cell communication between T/NK cells and other cells including monocytes, DCs, Treg, ILC1, and B subclusters (Supplementary Fig. S4e, f) in G-BM. Cell–cell ligand–receptor interaction analysis showed extensive communication between T/NK lineage and various potential sender cells, especially monocytes and DCs (Supplementary Fig. S5a). We then performed differential intercellular communication analysis of T and NK cells with their potential sender cells using scDiffCom^54^. In mobilized effector CD8^+^ T cells, upregulated ligand–receptor interactions contained pairs such as HLA-DQA1:LAG3 and LGALS3:LAG3 (Fig. 6a; Supplementary Table S9), which involve the immune checkpoint receptor gene *LAG3* with ability to regulate immune response^55^. In addition, upregulated interactions also contained ligand–receptor pairs potentially related to immunomodulation such as CTSG:F2R and TGFB1:CXCR4 (Fig. 6a)^56,57^. Moreover, these upregulated interactions can regulate the increased expression of downstream target genes related to immunosuppression and anti-inflammation (*TSC22D3*, *AREG*^58^ and *NFKB1A*^41^), further supporting their roles in immune regulation (Fig. 6b, c). Remarkably, another upregulated ligand–receptor pair S100A8:CD69, which was considered as an immunomodulation-related interaction, was enhanced in naïve CD4^+^ T cells, effector CD4^+^ T cells and certain effector CD8^+^ T cells (CD8_effector_RORC, CD8_effector_GZMH, and CD8_effector_CD69) with their sender cells (CD14^+^ monocytes, pre-monocytes, and intermediate monocytes) in G-BM (Fig. 6a; Supplementary Fig. S5b–g)^59^.

In mobilized NK cells, genes of upregulated ligand–receptor pairs were enriched in immunosuppressive pathways such as those associated with reduced NK cell-mediated cytotoxicity and negative regulation of immune effector process (Fig. 6d; Supplementary Fig. S6a, b and Table S9). These upregulated interactions in mobilized NK cells can further regulate similar downstream target genes as those in CD8^+^ T cells (Fig. 6e, f). In detail, the elevated ligand–receptor pairs included the interaction between type I MHC molecules and inhibitory killer cell immunoglobulin-like receptors (iKIRs) KIR2DL3, KIR3DL2 (HLA-A:KIR2DL3, HLA-A:KIR3DL2) (Fig. 6d), which were reported to play essential roles in NK cell activity^60^. In addition, it was worth noting that G-CSF increased GRN:TNFRSF1B interactions between NK cells and their sender cells (cDCs and pDCs) (Fig. 6d; Supplementary Fig. S6c–g). Interaction of PGRN (encoded by *GRN*, an antagonist of TNFα signaling) and TNFR2 (encoded by *TNFRSF1B*) has been reported to mediate anti-inflammation process in multiple inflammatory conditions^61,62^. Collectively, we comprehensively explored the potential indirect mechanisms by which G-CSF regulated immune response of T and NK cells via cell-to-cell communication.

## Discussion

G-CSF is a hematopoietic growth factor that induces granulopoiesis and HSPC mobilization. Our data showed a significantly increased percentage of myeloid cells, as well as that of HSPCs, especially GMP. A previous study also reported acceleration of GMP formation upon the release of G-CSF in mouse BM probably by triggering the *Irf8* and β-catenin progenitor self-renewal network in GMP^7^. However, the specific stages in which G-CSF induces enhancement of myelopoiesis and the underlying molecular mechanisms remain unclear, especially in humans. Combining the significantly changed percentages of GMP and lymphoid progenitors as well as the trajectory inference for hematopoiesis, LMPP was speculated as the earliest target progenitor of G-CSF inducing myeloid-biased hematopoiesis in human BM.

Besides, we further characterized the immunologic alterations in immune cells upon G-CSF administration. Consistent with the previous studies^18,63,64^, our data showed that CD4^+^ T cells tended to be Th2 polarized, with the downregulation of proinflammatory pathway-related cytokines (e.g., IFN-γ). T and NK cells in G-BM had stronger apoptosis properties. It has been reported that G-CSF enhanced the apoptosis of splenic T cells to inhibit inflammation and potentially induce immune tolerance in mice^65^. We also observed that CD8^+^ T and NK cells had lower cytotoxicity scores, which further indicated hyporesponsiveness of T and NK cells in G-BM.

To explore the underlying mechanisms of these particular immunologic changes, we initially investigated the potential direct mechanism wherein G-CSF regulates T and NK cells by binding with G-CSFR. Our results showed that the expression of *CSF3R* was slightly higher upon G-CSF treatment as previously reported^66^. We further speculated that G-CSF might directly regulate the target genes related to the immune response such as *SOCS1*. Notably, our recent work, which has been posted on *bioRxiv* and is now under review elsewhere, has validated that the upregulated SOCS1 in T cells inhibited T cell proliferation in vitro, thus potentially contributing to immune tolerance^38^. Apart from the direct effect on T/NK cells, G-CSF was generally recognized to regulate T and NK cell function in an indirect manner mediated by interacting cells, especially monocytes and DCs^67,68^. Through cell-to-cell communication analysis, we found that intercellular interactions of T/NK cells with monocytes and DCs have undergone significant changes upon G-CSF administration. Some ligand–receptor interaction pairs were significantly upregulated, including S100A8:CD69 and GRN:TNFRSF1B. Previous studies have demonstrated that S100A8:CD69 interaction was required for the upregulation of STAT3 signaling pathway, thus promoting Treg differentiation and probably inhibiting Th17 differentiation^59^. The expression of *Cd69* in Treg cells in mice was considered to be necessary to maintain immune tolerance, probably by affecting the secretion of factors such as IL-10^69,70^. Here, CD69 in CD8^+^ T cells also showed elevated expression and enhanced interaction with S100A8 from monocytes in G-BM, implying a presumed role in immunomodulation of CD8^+^ T cells. In addition, the binding of PGRN and TNFR2 (GRN:TNFRSF1B) was reported to exhibit anti-inflammatory properties by competitively interfering with TNF/TNFR-mediated inflammation^62^. Furthermore, experiments in mice indicated that PGRN could significantly protect Treg from a downregulation caused by TNFα, and regulate the function of effector T cells by inhibiting IFN-γ secretion^45,61^. Taken together, these ligand–receptor interactions may play complicated roles in cell-mediated regulation of immune response and inflammation in G-BM.

In summary, we provided a high-resolution transcriptome map of G-BM and deciphered the effects of G-CSF on lymphomyeloid divergence and immune hyporesponsiveness. Our results suggest that G-CSF-induced myeloid-biased differentiation initiates from the stage of LMPPs in human BM. Moreover, we delineated the potential cellular and molecular mechanisms of the immunosuppressive effect of G-CSF in human G-BM. G-CSF stimulation can upregulate the expression of genes related to immune hyporesponsiveness in T and NK cells via the direct binding with G-CSFR and diverse cell-to-cell communications. These findings will further provide implications for allo-HSCT and immune cell-based targeted therapy.

## Materials and methods

### Human samples

We recruited two healthy donors and treated them with recombinant G-CSF (filgrastim; Kirin Co., Ltd., Tokyo, Japan) at a dosage of 5 μg/kg body weight per day for 5 consecutive days. The BM samples were collected by aspiration on the fourth day of G-CSF treatment. The study was approved by the Ethics Committee of Peking University People’s Hospital and was conducted in accordance with The Declaration of Helsinki. Informed consents were obtained from all participants.

### FACS

Human BM cells were isolated from healthy donors before and after in vivo 5-day G-CSF application by Ficoll density centrifugation. Erythrocytes were removed by incubating with RBC lysis buffer (BD, 555899). After neutralization, the remaining cells were collected by spinning at 350× *g* for 6 min and suspended in FACS buffer (1× PBS with 1% BSA) for subsequent staining. Cells were stained in sorting buffer with specific antibodies for 30 min at 4 °C. The following antibodies were used for the staining and sorting: anti-CD45 (BV421, BD, 563879), anti-CD66b (PE, BD, 561650), alongside the viability dye 7-amino-actinomycin D (7-AAD) PerCP-Cy5.5 (eBioscience, 00699350). Given the dramatic increase in neutrophils after G-CSF treatment, we pooled neutrophils and other cells together in a certain ratio for scRNA-seq to construct a complete single-cell transcriptional landscape of human G-BM. Through flow sorting, the final cells used for scRNA-seq contained CD45^+^CD66b^–^ population (90%), CD45^+^CD66b^+^ population (5%), and CD45^–^ population (5%). Cells were sorted using an Aria II Flow Cytometer (BD Bioscience). Pre-gating was first done for live cells based on a 7-AAD staining. Data were analyzed using BD FACSDIVA v8.0.1 and Flowjo (v10).

### Single-cell RNA library preparation and sequencing

For 10× Genomics-based scRNA-seq, we implemented the Chromium Single Cell 3’ v2 libraries, under the guidance of the official instruction manual (https://support.10xgenomics.com/single-cell-gene-expression/library-prep/doc/technical-note-assay-scheme-and-configuration-of-chromium-single-cell-3-v2-libraries). Sequencing was performed on the Illumina Hiseq X Ten platform (Novogene, provided by Berry Genomics Corporation, Beijing, China).

### scRNA-seq data processing

For scRNA-seq data, raw gene expression matrices were generated for each sample by the Cell Ranger (version 4.0.0) pipeline coupled with human reference hg19. The output gene expression matrices were analyzed by R software (version 3.6.3) with the Seurat package (version 3.2.2)^71^. Low-quality cells were removed if they met any of the following criteria: (1) < 1000 unique molecular identifiers (UMI); (2) < 300 genes; or (3) > 10% UMIs derived from the mitochondrial genome. Doublets were identified by the R package DoubletFinder^72^ with default settings. After the removal of the low-quality cells and doublets, a total of 21,005 cells and 26,937 genes were kept for further analyses. Then, UMI counts were normalized by the NormalizeData function and top 2000 features with high cell-to-cell variation were calculated using the FindVariableFeatures function. Next, the ScaleData function was conducted to scale and center features in the datasets and the RunPCA function was used with default parameters to reduce dimensionality. The R package Harmony (version 0.1.0)^73^ was used for batch correction to avoid the batch effect across samples. The data were used in the subsequent nonlinear dimensional reduction with the RunUMAP function and cluster analysis by the FindNeighbors and FindClusters functions. All details regarding the Seurat analyses performed in this work can be found in the website tutorial (https://satijalab.org/seurat/v3.0/pbmc3k_tutorial.html).

### Cell type annotation and cluster marker identification

After the projection of all cells into two-dimensional spaces by UMAP, cells were clustered together according to common features. The annotation of cell clusters was identified by the expression of cell type-associated genes. SCENIC^74^ was also used to help the cell state identification based on key regulon activity. The regulon activity scores were calculated with AUC by the AUCell package. Signature genes of each cell cluster were identified with the FindAllMarkers function in Seurat. To further decode the hematopoietic landscape in G-BM, major cell types were further sub-clustered. Scaling, principal component analysis, and clustering were performed as described above. Additionally, all percentages of different cell types were calculated after removing neutrophils.

### Differential gene expression and functional enrichment

DEGs were recognized using the FindAllMarkers function in Seurat with parameter ‘test.use = wilcox’ by default and the Benjamini–Hochberg method was used to estimate the false discovery rate (FDR). DEGs were filtered by fold change of > 1.25. DEGs with *P* value adjusted by Benjamini–Hochberg < 0.05 were considered significant. Enrichment analysis of DEGs was conducted using the Metascape webtool (https://metascape.org). GSEA was performed using the clusterProfiler package (v3.13.0)^75^.

### Trajectory inference

To map hematopoietic differentiation, pseudotime analysis was performed with Monocle v2. For the pseudotime ordering analyses (before and after G-CSF administration), the gene expression matrix issued from the integrated samples was loaded in Monocle2 using the new CellData function. The filtered genes with mean expression ≥ 0.1 and an empirical dispersion at least twice as large as the fitted dispersion were set as ordering genes. To avoid cell cycle effects, cell cycle genes in GO: 0007049 were further excluded from ordering genes. Then, ‘DDRTree’ function was used for dimensionality reduction and ‘plot_cell_trajectory’ function for visualization.

### Defining cell state scores and cell cycle analysis

AddModuleScore function in Seurat package was used to calculate the gene set scores. We used cell proliferation (GO: 0007049), leukocyte migration (GO: 0050900), positive regulation of apoptosis process (GO: 0043065), an immnuosuppression-related gene set (61 genes)^76^, 4 cytotoxicity-associated genes (*GZMA*, *GZMB*, *KLRD1,* and *NKG7*) and 829 highly expressed genes reported in M-MDSCs^52^ to define proliferation, migration, apoptosis, immunosuppression, cytotoxicity, and M-MDSC scores, respectively. For cell cycle analysis, CellCycleScoring function in Seurat package was used to predict the classification of each cell in either G1, S or G2M phase.

### Cell–cell communication analysis

Ligand–receptor interactions were identified by CellPhoneDB^77^ with default parameters. Only ligand–receptor interaction pairs with *P* value < 0.05 returned by CellPhoneDB were considered significant. Scdiffcom^54^ was used to perform differential intercellular communication analysis before and after G-CSF administration. Up- or downregulated ligand–receptor pairs were then filtered with adjusted *P* value < 0.05 and fold change > 1.5. The regulatory effect of the differential communication on T and NK cells was further explored by predicting downstream target genes of curated ligand–receptor interactions. To speculate target genes of altered ligand–receptor pairs, we retrieved ligand–receptor–target relationship from publicly available resources: intercellular links (ligand–receptor interactions) and intracellular subnetworks (receptor–transcriptional factor pathways and ligand–target links) from NicheNet^40^ and scMLnet^78^ for further analysis. Only DEGs before and after G-CSF administration were included into analysis of target gene prediction. The igraph R package and Cytoscape (version 3.6.1) were used for visualization of the ligand–receptor–target relationship.

### Statistical analysis

Wilcoxon-rank-sum test and Student’s *t*-test were used for comparisons of gene expression levels. We considered *P* value < 0.05 statistically significant. Analyses were conducted using R software (version 3.6.3).

## Supplementary information

Supplementary Information

Supplementary Table S1

Supplementary Table S2

Supplementary Table S3

Supplementary Table S4

Supplementary Table S5

Supplementary Table S6

Supplementary Table S7

Supplementary Table S8

Supplementary Table S9

## Data Availability

The scRNA-seq data of our study have been deposited at GEO (NCBI) with accession number GSE193138.
