# Supplementary material for: Decoding lymphomyeloid divergence and immune hyporesponsiveness in G-CSF-primed human bone marrow by single-cell RNA-seq
Source: Cell Discov. 2022 Jun 22;8:59. doi: 10.1038/s41421-022-00417-y (PMC9217915; doi:10.1038/s41421-022-00417-y)
Supplement: Supplementary file 1 — Supplementary Information [file 41421_2022_417_MOESM1_ESM.pdf]

Supplementary Figure S1

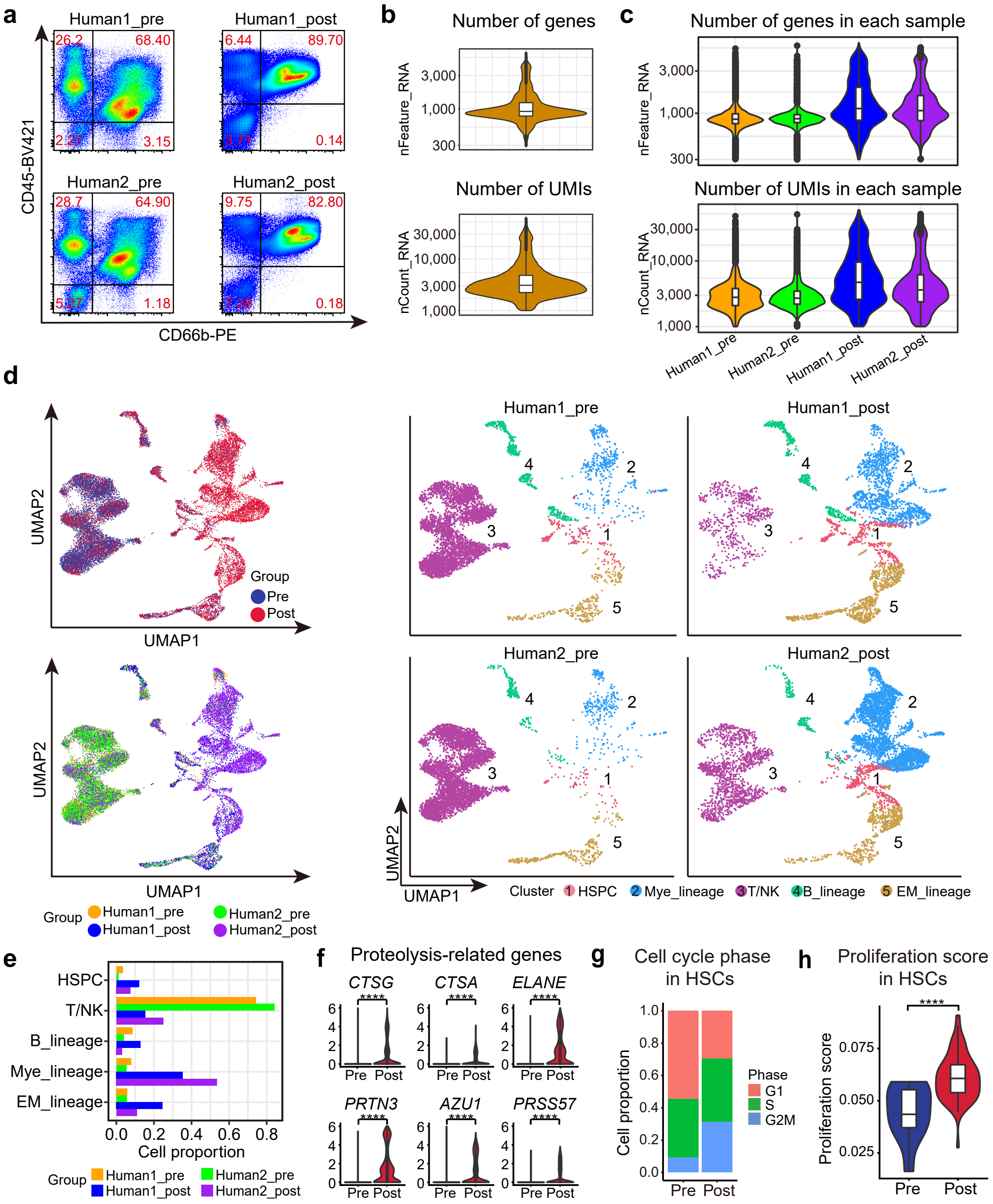

Supplementary Fig. S1. Overview of quality of scRNA-seq data and characteristics of hematopoietic cells in G-BM.

a Sorting strategy of hematopoietic cells for scRNA-seq. **b** Violin plots showing average gene and unique molecular identifier (UMI) numbers of scRNA-seq across two donors. For box plot within each violin plot, center black lines indicate median values, boxes range from 25th to 75th percentiles. **c** Violin plots of average gene and UMI numbers of scRNA-seq across four samples from two donors before and after G-CSF treatment. **d** Uniform manifold approximation and projection (UMAP) visualization of hematopoietic cells in human bone marrow. Each dot represents a single cell; colors indicate cell clusters with sample stages (top) and sources of donors (bottom and right). HSPC, hematopoietic stem and progenitor cell; mye-lineage, myeloid lineage; EM_lineage, Erythroid/megakaryocyte lineage. Pre, representing samples before G-CSF treatment; Post, representing samples after G-CSF treatment. **e** Bar plot showing the changes in the percentage of cell lineages upon G-CSF treatment across four samples from two donors. **f** Violin plots showing the expression of genes related to proteolytic environment in hematopoietic cells in human bone marrow before and after G-CSF treatment. P value < 0.05 is considered to be statistically significant. **** P value < 0.0001. **g** Stacked bar chart showing the constitution of different cell cycle phases of HSCs before and after G-CSF treatment based on the average expression of S and G2/M gene sets. **h** Violin plot showing the proliferation score in HSCs before and after G-CSF treatment. Wilcoxon Rank Sum test is employed to test the significance of difference and the P value is indicated for the comparison. **** P value < 0.0001.

Supplementary Figure S2

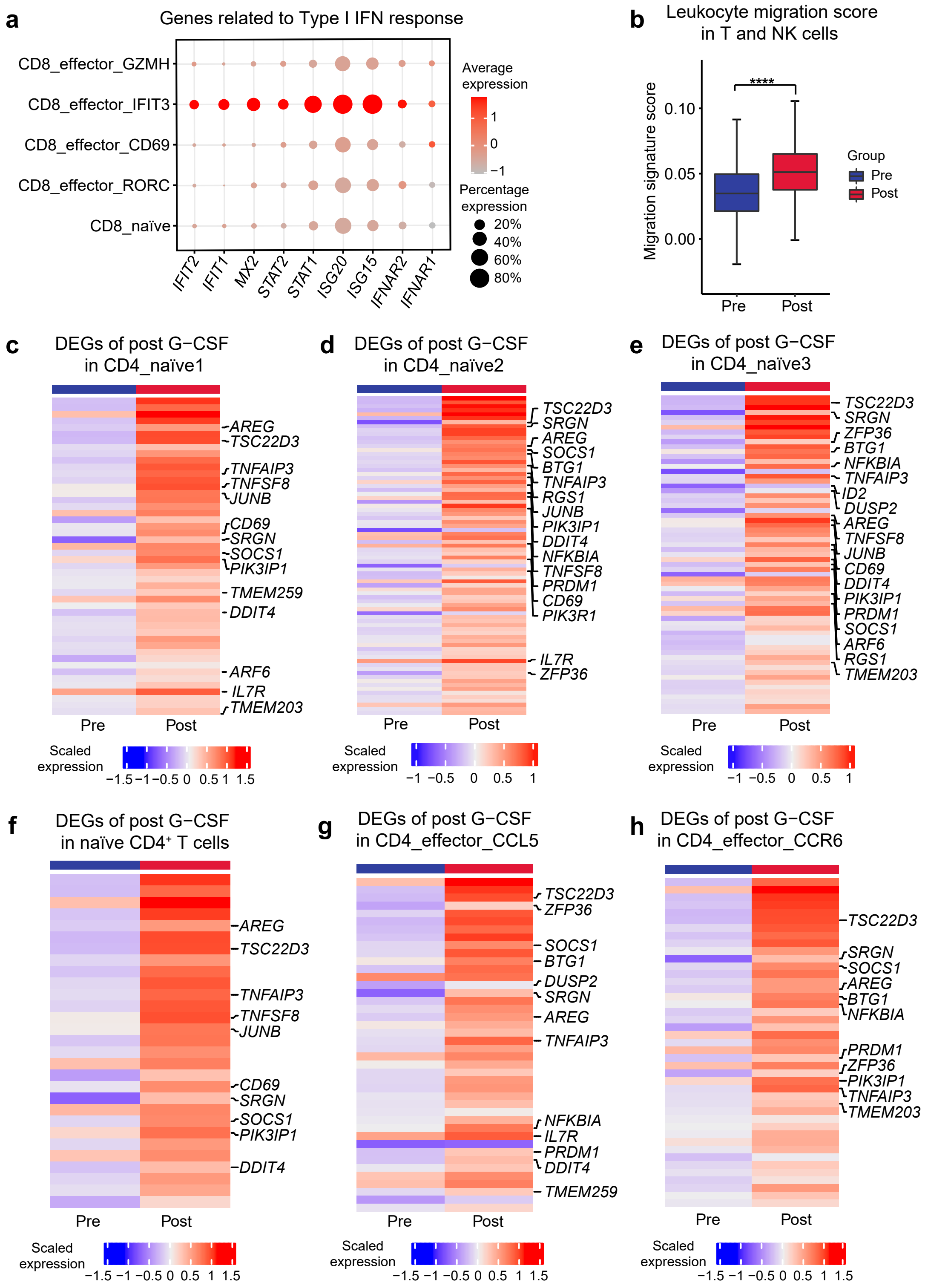

Supplementary Fig. S2. The impact of G-CSF on the T/NK lineage in human bone marrow.

a Dot plot showing average expression and percentage of expressed cells of genes related to type I IFN receptors and type I IFN response in each $CD8^+$ T subcluster. **b** Box plot of the leukocyte migration score in the T/NK lineage before and after G-CSF administration. **** P value < 0.0001. **c-h** Heatmaps displaying scaled expression of differentially expressed genes (DEGs) highly expressed after G-CSF administration in $CD4^+$ T subclusters including CD4_naïve1 (**c**), CD4_naïve2 (**d**), CD4_naïve3 (**e**), overall naïve $CD4^+$ T cells (**f**), CD4_effector_CCL5 (**g**) and CD4_effector_CCR6 (**h**).

Supplementary Figure S3

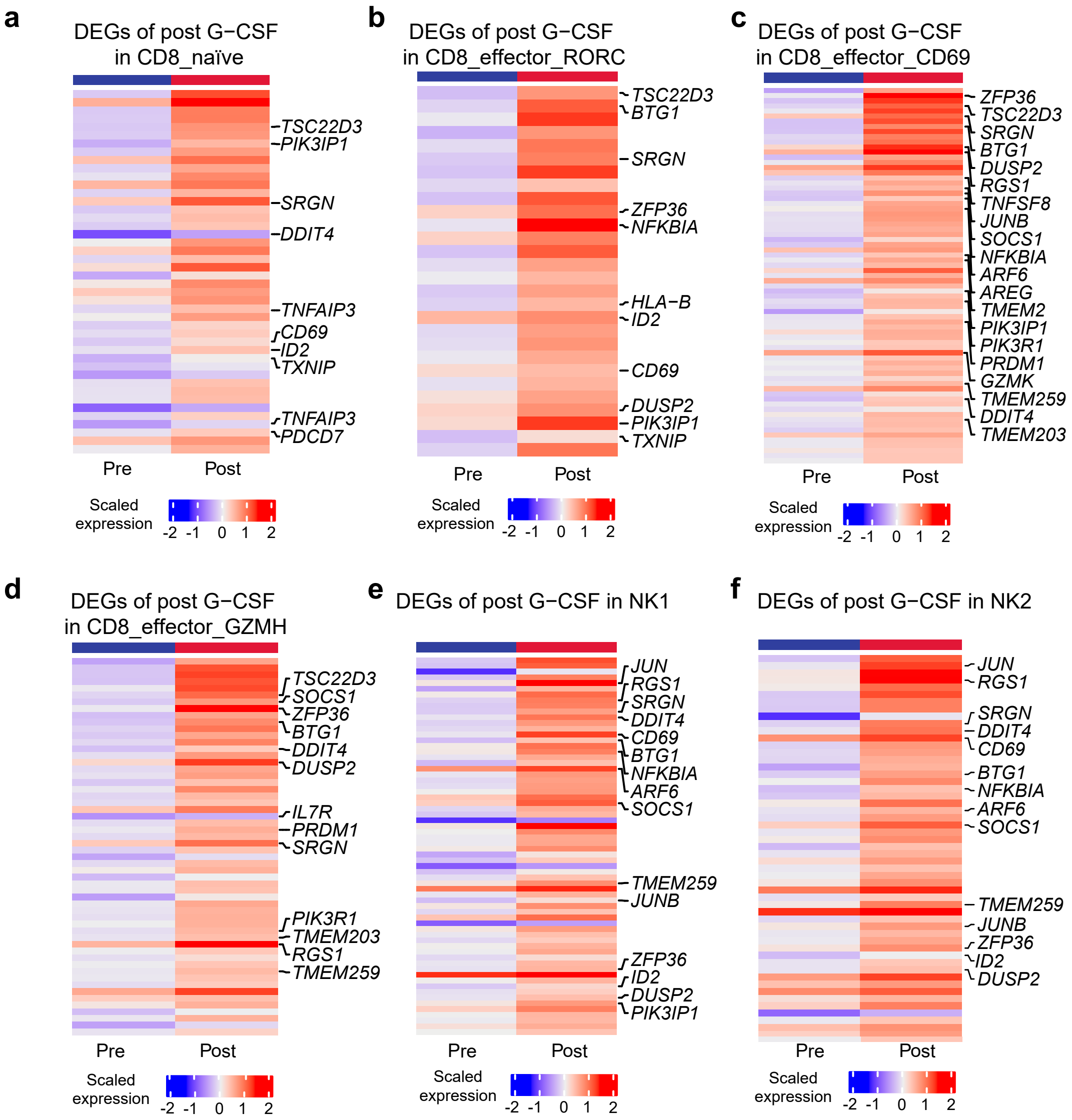

Supplementary Fig. S3. Differentially up-regulated genes in G-BM of $CD8^+$ T and NK cell subclusters.

a-d Heatmaps displaying scaled expression of DEGs highly expressed after G-CSF administration in $CD8^+$ T cell subclusters including CD8_naïve (**a**), CD8_effector_RORC (**b**), CD8_effector_CD69 (**c**), CD8_effector_GZMH (**d**). **e-f** Heatmaps displaying scaled expression of DEGs highly expressed after G-CSF administration in NK cell subclusters including NK1 (**e**) and NK2 (**f**).

Supplementary Figure S4

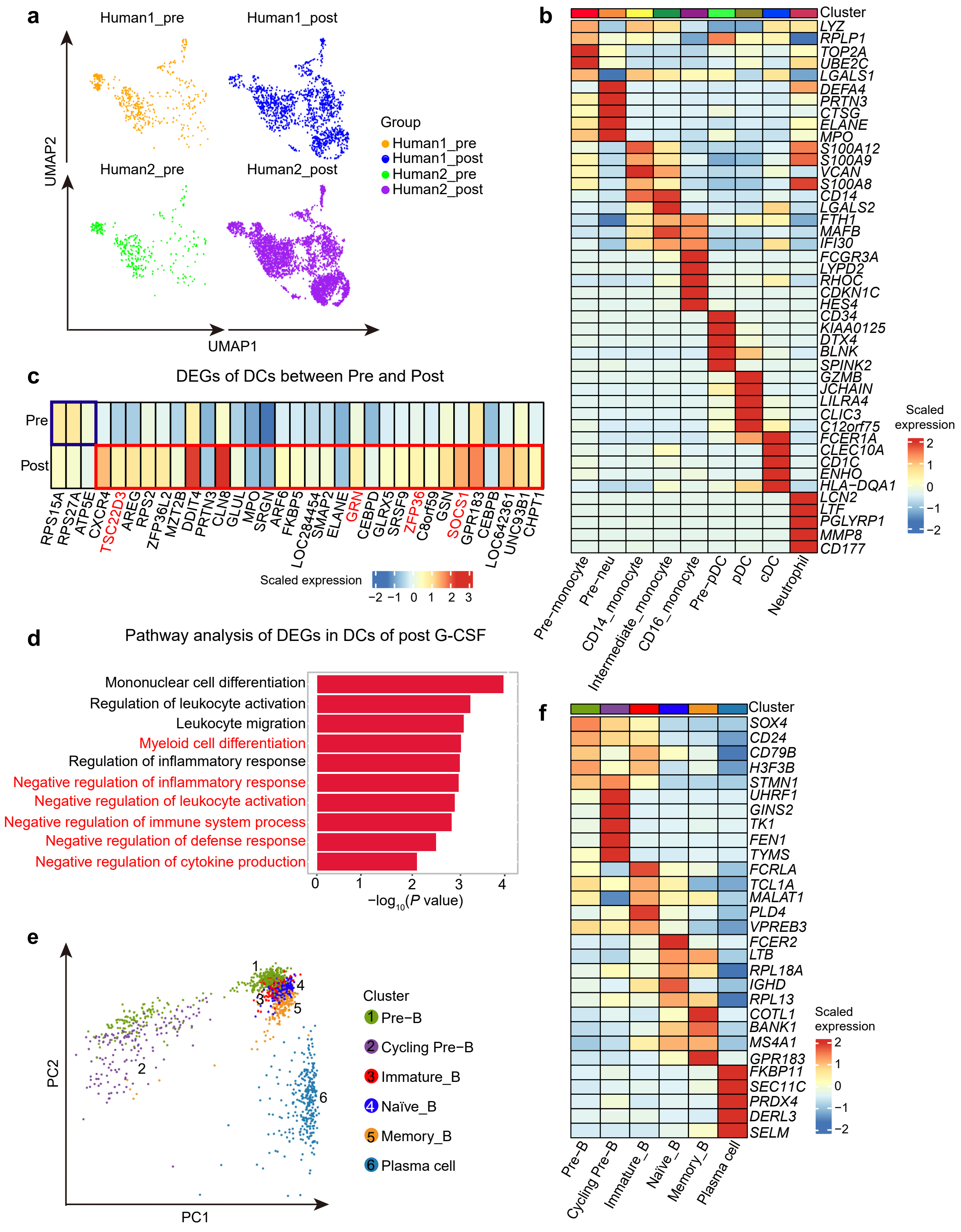

Supplementary Fig. S4. The effect of G-CSF on myeloid cells and sub-clustering of B lineage.

a UMAP visualization of myeloid lineage distribution from each sample before and after G-CSF administration across two donors. **b** Heatmap showing scaled expression of top 5 DEGs of myeloid subclusters. Detailed DEGs can be found in Supplementary Table S7. **c** Heatmap showing average expression of top DEGs in dendritic cells (DCs) before and after G-CSF administration. Detailed DEGs can be found in Supplementary Table S8. **d** Bar plot from the Metascape analysis showing the representative enrichment terms of significantly up-regulated genes in DCs upon G-CSF administration. The length of each bar represents $-\log_{10}$ (P value). **e** UMAP visualization of 6 subclusters of B lineage. **f** Heatmap showing scaled expression of top 5 DEGs of each subcluster in B lineage.

Supplementary Figure S5

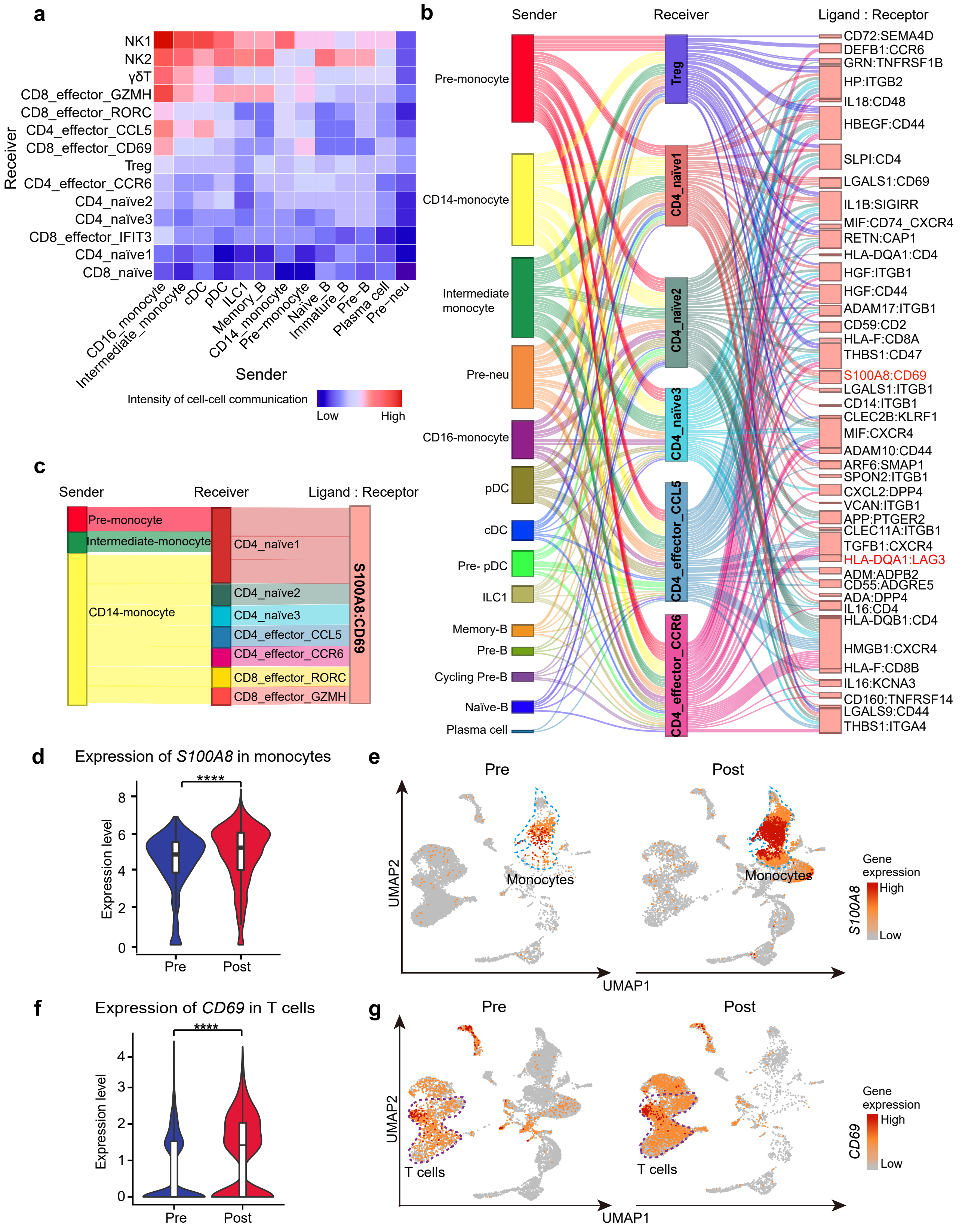

Supplementary Fig. S5. Effect of G-CSF on cell-cell communication in T cells.

a Heatmap showing the number of potential interactions predicted by CellPhoneDB between T and NK cells and their potential sender cells, where intensity of red color is proportional to the number of the ligand-receptor pairs. Only the interactions with P value < 0.05 were selected. **b** Sankey plot showing the up-regulated intercellular communication between the potential sender cells and $CD4^+$ T cells after G-CSF administration compared with unstimulated BM. Differential intercellular communication analysis was performed using R package scDiffCom. Ligand-receptor pairs with adjusted P value < 0.05 and fold change > 1.5 are displayed. **c** Sankey plot showing S100A8: CD69 ligand-receptor interaction between T cells (naïve $CD4^+$ T cells, effector $CD4^+$ T cells, CD8_effector_RORC, CD8_effector_GZMH and CD8_effector_CD69) and their sender cells (Pre-monocytes, intermediate monocytes and $CD14^+$ monocytes). **d** Violin plot showing the expression of *S100A8* in monocytes (Pre-monocytes, intermediate monocytes and $CD14^+$ monocytes). **** P value < 0.0001. **e** UMAP visualization of expression patterns of *S100A8* in hematopoietic cells before and after G-CSF administration. **f** Violin plot showing the expression of *CD69* in T cells including naïve $CD4^+$ T cells, effector $CD4^+$ T cells, CD8_effector_RORC, CD8_effector_GZMH and CD8_effector_CD69. **** P value < 0.0001. **g** UMAP visualization of expression patterns of *CD69* in hematopoietic cells before and after G-CSF administration.

Supplementary Figure S6

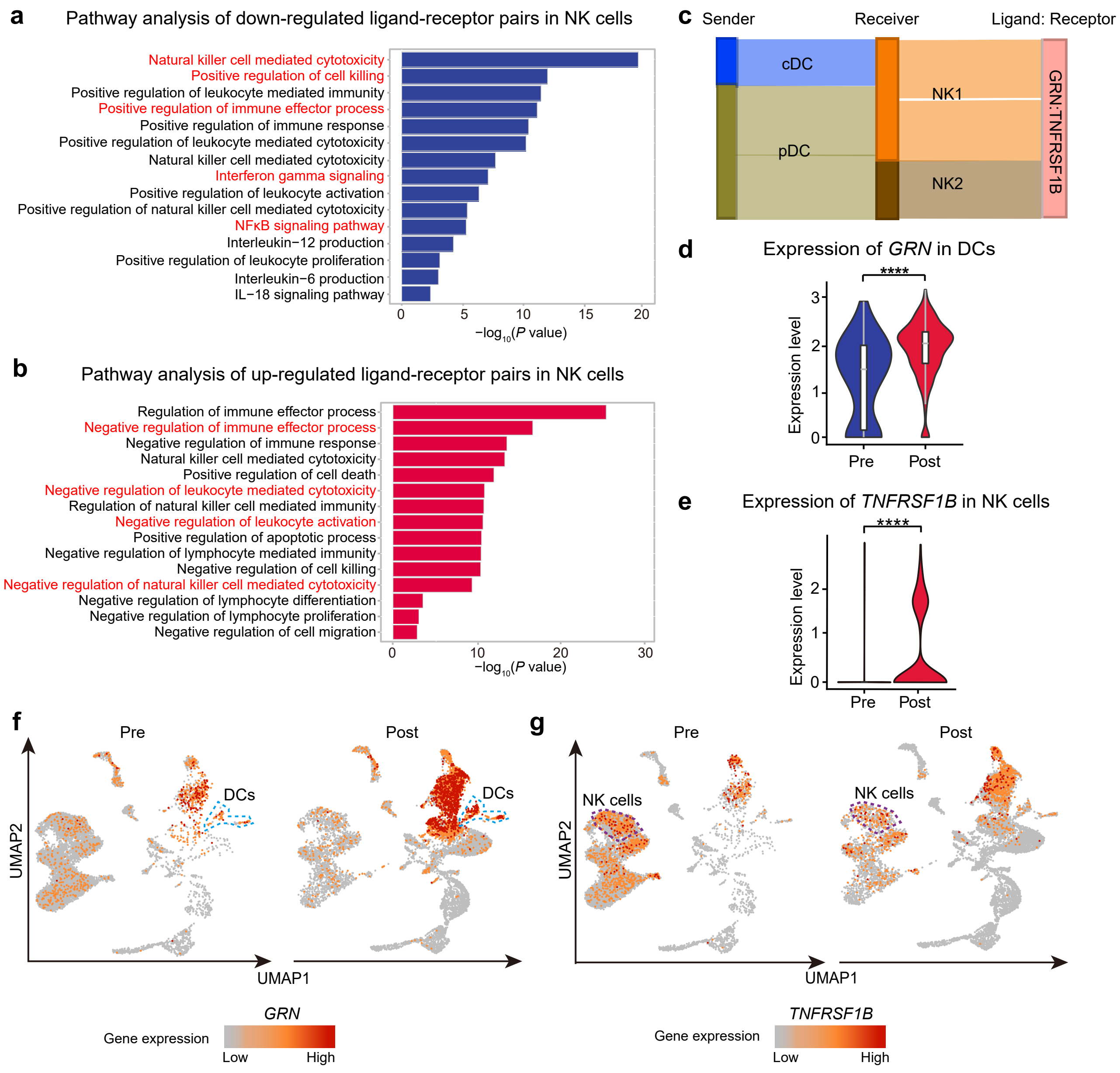

Supplementary Fig. S6. Effect of G-CSF on cell-cell communication in NK cells.

a Bar plot from the Metascape analysis showing the representative terms of genes in significantly down-regulated ligand-receptor pairs in NK cells upon G-CSF administration. The length of each bar represents $-\log_{10}$ (*P* value). **b** Bar plot from the Metascape analysis showing the representative terms of genes in significantly up-regulated ligand-receptor pairs in NK cells upon G-CSF administration. The length of each bar represents $-\log_{10}$ (*P* value). **c** Sankey plot showing GRN: TNFRSF1B ligand-receptor interaction between NK cells and their sender cells (cDCs and pDCs). **d** Violin plot showing the expression of *GRN* in DCs (cDCs and pDCs). **** *P* value < 0.0001. **e** Violin plot showing the expression of *TNFRSF1B* in NK cells before and after G-CSF administration. **** *P* value < 0.0001. **f** UMAP visualization of expression patterns of *GRN* in hematopoietic cells before and after G-CSF administration. **g** UMAP visualization of expression patterns of *TNFRSF1B* in hematopoietic cells before and after G-CSF administration.
